# Exosomes Derived from AT2R-Overexpressing BMSC Prevent Restenosis After Carotid Artery Injury by Attenuating the Injury-Induced Neointimal Hyperplasia

**DOI:** 10.1007/s12265-022-10293-2

**Published:** 2022-07-28

**Authors:** Xinliang Zou, Yi Liao, Zhihui Liu, Xiang Xu, Weiwei Sun, Haoran Qin, Haidong Wang, Jianping Liu, Tao Jing

**Affiliations:** 1 Department of Cardiology, Southwest Hospital, Army Medical University, Chongqing, 400038 People’s Republic of China; 2 Department of Thoracic Surgery, Southwest Hospital, Army Medical University, Chongqing, 400038 People’s Republic of China

**Keywords:** AT2R, Exosomes, Carotid artery, Neointimal hyperplasia, Restenosis

## Abstract

Restenosis is a severe complication after percutaneous transluminal coronary angioplasty which limits the long-term efficacy of the intervention. In this study, we investigated the efficiency of exosomes derived from AT2R-overexpressing bone mesenchymal stem cells on the prevention of restenosis after carotid artery injury. Our data showed that AT2R-EXO promoted the proliferation and migration of vascular endothelial cells and maintained the ratio of eNOS/iNOS. On the contrary, AT2R-EXO inhibited the proliferation and migration of vascular smooth muscle cells. In vivo study proved that AT2R-Exo were more effectively accumulated in the injured carotid artery than EXO and Vehicle-EXO controls. AT2R-EXO treatment could improve blood flow of the injured carotid artery site more effectively. Further analysis revealed that AT2REXO prevents restenosis after carotid artery injury by attenuating the injury-induced neointimal hyperplasia. Our study provides a novel and more efficient exosome for the treatment of restenosis diseases after intervention.

**Supplementary Information:**

The online version contains supplementary material available at 10.1007/s12265-022-10293-2.

## Introduction

Coronary artery disease is a common threat to human health which is also a prominent death-causing cardiovascular disease [1]. Despite numerous improvements in technical equipment and surgical materials, studies have shown in stent restenosis within 8 to 12 months after surgery-induced mechanical injuries is the most primary pathological issue which limits the prognoses of patients [2, 3]. It is well known that the main cause of stent restenosis is neointimal hyperplasia (NIH), which is strongly associated with the differentiation, proliferation, and migration of vascular endothelial cells (VECs) and smooth muscle cells (VSMCs) [4, 5]. However, because of the lack of clear understanding of the molecular mechanism of the formation of neointima, the treatment of restenosis is still far from satisfactory.

Studies have shown that the biological effects of angiotensin II type 2 receptor (AT2R) is antagonistic to the effects of angiotensin II type1 receptor (AT1R), which is negatively correlated with the vascular tension, cell proliferation and migration [6, 7]. Our previous study has shown that the overexpression of AT2R significantly reduced the neointimal hyperplasia of carotid artery after percutaneous coronary intervention in rats [8]. Since the expression of AT1R is maintained at a high-level during life-time and further increased after vascular injury, while the expression of AT2R only slightly reappeared after vascular injury. Therefore, enhancing the expression of AT2R in injured vessels may be beneficial to the prevention and treatment of restenosis.

Exosomes are extracellular tiny cell membrane vesicles with a diameter of 30–150 nm and cargoes of lipids, proteins, RNA, miRNAs, and mRNAs [9, 10]. By fusing with the recipient cells and releasing the cargoes, exosomes could influence their function and physiology. Therefore, they are considered as the crosstalk factors in intercellular communication [11, 12]. Recently, exosomes have been confirmed with a variety of repair functions. For example, M2 macrophage-derived exosomes soften nearby VSMCs, hence accelerating the vascular tissue repair process after intravascular stent implantation [12]. Our previous study also reported that bone mesenchymal stem cells (BMSCs)-derived exosomes could suppress NIH after artery injury by activating the Erk1/2 signaling pathway [13]. Nevertheless, there is still no clear conclusion on how exosomes affect the behavior of VSMCs and VECs during the repair after stent implantation. Also, whether the exosomes derived from AT2R-overexpressing BMSCs play a role in the angiogenesis is still unknown.

In the present study, we hypothesized that exosomes derived from AT2R-overexpressing BMSC could more effectively promote the activation of vascular endothelial cells after vascular damage to prevent restenosis. We aim to investigate the role of exosomes derived from AT2R-overexpressing BMSCs in restoring vascular function after damage and to clarify the mechanism of these exosomes in prevent NIH and providing new ideas for the treatments and prevention of restenosis.

## Materials and Methods

### Cell Collection and Culture

The bone marrow cells were collected form the medullary cavity of tibia and femur of four-week-old SD rats and cultured in MSCM medium supported with 5% exosome-depleted fetal bovine serum (FBS), 1% mesenchymal stem cell growth supplement, and 1% penicillin/streptomycin (P/S) Solution (7501, Sciencell, USA) at 37 °C with 5% CO_2_. Then CD29 (FITC-labeled), CD90 (PE-labeled) positive, and CD11b negative (eFluor® 450-labeled) cells were further isolated by flow cytometry analysis. The rat vascular (carotid artery) endothelial cells and smooth muscle cells were cultured in 1640 medium (L220KJ, Basalmedia, Shanghai, China) supported with exosome-free 10% FBS (SV30087, Hyclone, America) and 1% P/S solution (S110JV, Basalmedia, Shanghai, China) at 37 °C with 5% CO_2_.

## Isolation and Characterization of Mesenchymal Stem Cell-Derived Exosomes

After 72 h of culture with Monensin (4 µM: M8670, Solarbio, Beijing, China), exosomes were isolated from the BMSCs culture supernatant by using the Exo Quick‐TC Kit (EXOTC50A-1, System Biosciences, USA) according to the manufacturer’s instructions. Then, exosomes were further assessed by transmission electron microscopy, nanoparticle tracking analysis and immunoblotting analysis of exosomal markers.

### Cell Viability Assay

The cell counting kit-8 (CCK-8, CA1210, Solaribio, Beijing, China) was used to assess the cell viability of VECs and VSMCs. Briefly, cells were seeded at 1 × 10^3^ cells/well into a 24-well plate overnight. Then cells were treated with hypoxia (1% O_2_, 5% CO_2_, 94% N_2_) and with 125 ng/mL, 250 ng/mL, 500 ng/mL, and 1000 ng/mL of indicated exosomes for 24 h and 48 h. At each time point, 30 µL of CCK-8 reagent was added into each well and cultured for 2 h in dark. The absorbance was measured at 450 nm by using Microplate Reader.

### TUNEL Assay and Measurement NO Level

VECs and VSMCs were seeded at 5 × 10^4^ cells/well into a 6-well plate overnight. Then VECs were treated with hypoxia (1% O_2_, 5% CO_2_, 94% N_2_) and with 250 ng/mL of indicated exosomes for 48 h. VSMCs were treated with hypoxia (1% O_2_, 5% CO_2_, 94% N_2_) and with 500 ng/mL of indicated exosomes for 48 h. Then, cell apoptosis was detected by using TUNEL assay kit (ab206386, Abcam) according to the manufacturer’s instructions. The NO level in the VECs supernatant samples were analysed by using an enzyme-linked immunosorbent assay kit (Shanghai Yueyan Biological Technology Co., Ltd, China) according to the manufacturer’s instructions.

### Cell Migration Assay

The HTS Transwell®-24-well Permeable Support with 8.0-μm pore polyester membrane and 6.5 mm Inserts (#3422, Corning, USA) was used for migration assay. Briefly, 1 × 10^4^ cells were seeded in the upper transwell chamber in 200µL serum-free medium. Fuel the lower chamber with 500µL culture medium and 250 ng/mL of indicated exosomes for VECs or 500 ng/mL of indicated exosomes for VSMCs, and treat with hypoxia (1% O_2_, 5% CO_2_, 94% N_2_) for 48 h. Then, cells adherent on the undersurface of the membrane were fixed with 4% PFA and stained by 0.05% crystal violet. Images of five random fields under inverted microscope were taken for each chamber to count the the number of migrated cells.

### Immunoblotting

Cells were lysed in ice-cold NP-40 buffer (N8032, Solaribio, Beijing, China) with the protease/phosphatase inhibitor cocktail (KGP2100, Keygen, China). A total of 30 µg protein of each sample were resolved by Precast-Gel (PG01210-S, Solarbio, Beijing, China) and transferred to nitrocellulose membranes (P-N66485, Solarbio, Beijing, China). After blocking with 5% skim milk for 1 h, the membrane was incubated with primary antibodies overnight at 4℃: anti-CD9 (1:1000, 98,327, Cell Signaling Technology, USA), anti-CD63 (1:1000, 25,682–1-AP, Proteintech, USA), anti-CD81 (1:1000, 66,866–1-Ig, Proteintech, USA), anti-HSP70 (1:1000, 66,183–1-Ig, Proteintech, USA), anti-Calnexin (1:1000, 10,427–2-AP, Proteintech, USA), anti-AT2R (1:1000, ab92445, abcam, UK), anti-eNOS (1:1000, 27,120–1-AP, Proteintech, USA), anti-iNOS (1:1000, 18,985–1-AP, Proteintech, USA), anti-β-actin (1:1000, 66,009–1-Ig, Proteintech, USA), anti-CTGF (1:1000, 23,936–1-AP, Proteintech, USA), anti-TGF-β1 (1:1000, 21,898–1-AP, Proteintech, USA), anti-α-SMA (1:1000, 19,245, Cell Signaling Technology, USA), anti-SM-MHC (1:1000, 21,404–1-AP, Proteintech, USA), anti-Luciferase (1:1000, ab185926, abcam, UK), anti-IL-1β (1:1000, 16,806–1-AP, Proteintech, USA), anti-TNF-α (1:1000, 17,590–1-AP, Proteintech, USA). After wash, the membrane was incubation with corresponding horseradish peroxidase-conjugated secondary antibodies for 1 h, and developed by using SuperSignal™ West Pico PLUS Chemiluminescent Substrate (34,580, Thermo Scientific, MA, USA).

### ELISA Assay

1 × 10^6^ VECs were seeded in each well of 6-well plate and incubate overnight. Then cells were treated with hypoxia (1% O_2_, 5% CO_2_, 94% N_2_) and with 125 ng/mL, 250 ng/mL, 500 ng/mL, and 1000 ng/mL of indicated exosomes for 24 h and 48 h. The supernatants were collected. ELISA assay for secretion of TNF-α and IL-1β were performed using human cytokine ELISA Kit (ab181421 and ab214025, abcam, UK) according to manufacturer instructions.

### Animal Experiments

The construction of animal models of carotid artery injury in rats has been described in detail in our previously study [13]. The rats were randomly divided into sham operation, normal saline, Exo, Vehicle-EXO, and AT2R-EXO groups *(n* = 20/group). For the exosome groups, 0.5 mL of 200 µg/mL exosomes were intravenously injected every 3 days. The normal saline group received the same volume of normal saline. After 2 weeks of treatment, ten rats were sacrificed in each group. The carotid arteries of 5 rats were used for intravital imaging, and the carotid arteries of other 5 rats were used in subsequent analyses. The remaining rats were treated for another 2 weeks. During the treatment, the blood flow of the injured carotid artery site was measured by the spectrum Doppler.

### H&E, Masson Staining, Immunohistochemistry, and Immunofluorescence

Five rats of each group were sacrificed on weeks 2 and 4 after the treatment. The injured carotid arteries were harvested and fixed in 4% paraformaldehyde. Then, carotid artery samples were dehydrated and embedded in paraffin. The samples were sectioned into 6-µm-thick pieces, followed by staining with hematoxylin and eosin (H&E) and Masson staining. The Image-Pro Plus software program (Media Cybernetics, Rockville, MD) was used to measure the intimal to medial area ratio (I/M), neointimal area, media area, and collagen of the injured carotid arteries. For immunohistochemistry, the sections were deparaffinized, blocked and incubated with anti-CTGF (1:200, 23,936–1-AP, Proteintech, USA), anti-TGF-β1 (1: 200, 21,898–1-AP, Proteintech, USA), anti-α-SMA (1: 200, 19,245, Cell Signaling Technology, USA). For immunofluorescence, frozen sections were punched by 1% Triton X-100 (9002–93-1, Solarbio, Beijing, China), blocked, and incubated with anti-eNOS (1:300, 27,120–1-AP, Proteintech, USA) and anti-AT2R (1:250, ab92445, Abcam, UK).

### Zymography Assay

The zymography assay kit (P1700, Apply.gen, Beijing, China) was used to assess the activities of MMP2 and MMP9. Briefly, injured carotid arteries were ground in liquid nitrogen and lysed with 5 times the volume of lysis buffer (R0010 Solaribio, Beijing, China) for 30 min in ice bath. A total of 50 µg protein of each sample was resolved by 10% SDS-PAGE which was supported with gelatin (10 mg/mL). Then the PAGE gel was washed with buffer (2.5% Triton X-100, 50 mmol/L Tris–HCl, 5 mmol/L CaCl2, pH7.6) twice for 40 min/each, and further washed with buffer (50 mmol/L Tris-HC, 5 mmol/L CaCl2) twice for 20 min/each. Next, the gel was incubated in buffer (50 mmol/L Tris, pH7.5, 150 mmol/L NaCl, 10 mmol/L CaCl_2_, 0.02%Brij-35) at 37℃ for 4 h. Then, the gel was further stained with Commassie blue staining solution.

### Statistical Analysis

In our study, the Student’s *t* test was used to compare the difference between two groups. The ANOVA and Tukey post hoc tests of pairwise differences (Tukey HSD) were used to determine the difference of multiple groups. The statistical analysis was performed using SPSS 19.0 software, data are shown as the means ± SD. Difference with a P value less than 0.05 was considered significant.

## Results

### The Identification of ART2-Contained Exosomes

**Fig. 1:**
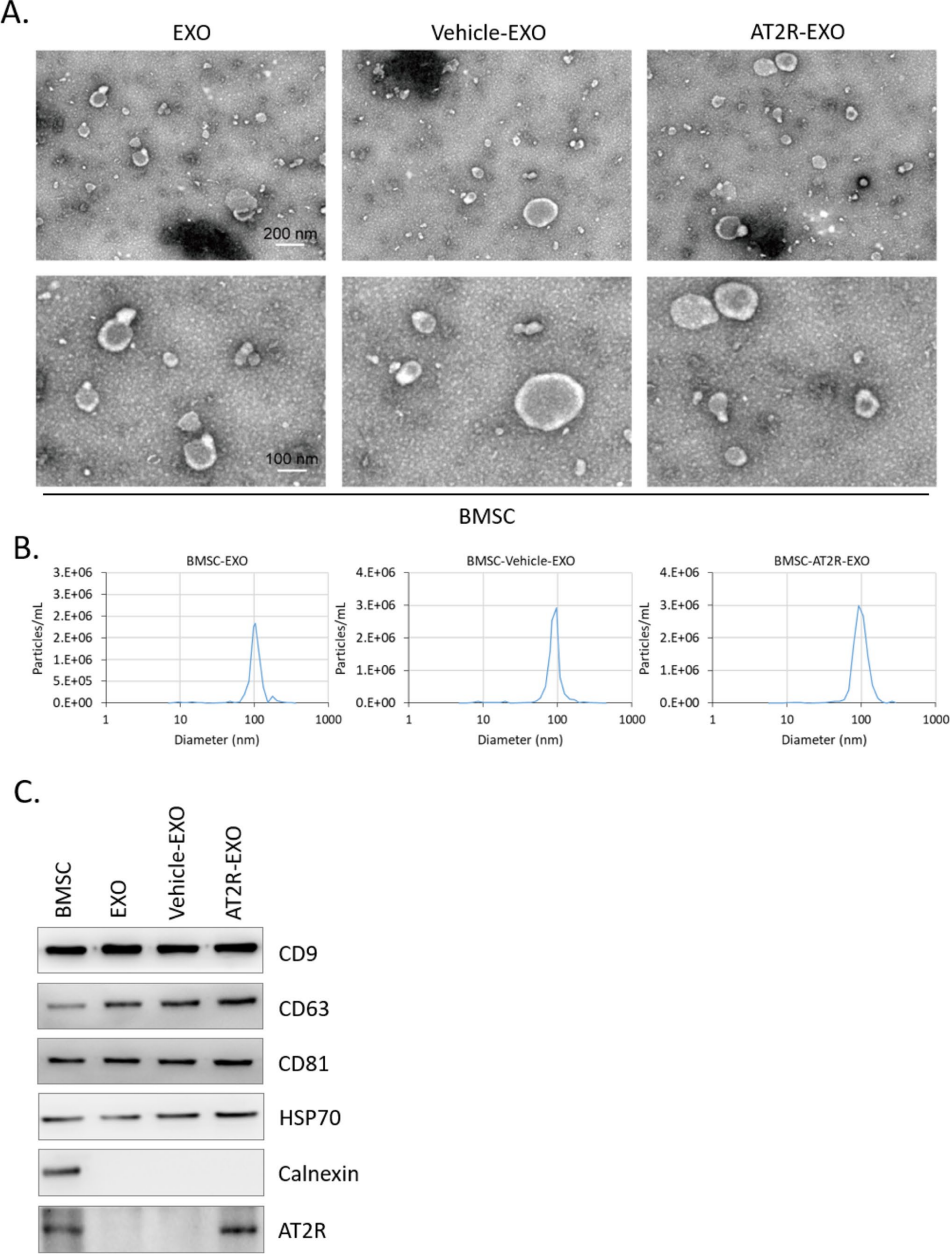
The identification of ART2-contained exosomes. **A** Representative transmission electron microscopy images of our isolated exosomes. **B** Nanoparticle tracking analysis of the size of the isolated particles. **C** Immunoblotting analysis of the expression of exosomal markers and AT2R in our isolated exosomes

Transmission electron microscopy showed the round-shaped membrane vesicles of our isolation (Fig. 1A). Particle size analysis revealed that the size of the isolated particles ranged from 70 to 150 nm (Fig. 1B). Further, the exosome-associated protein markers were detected by immunoblotting. Our data show that BMSC lysis control and all exosome particles expressed CD9, CD63, CD81, and HSP70, while calnexin was only expressed in BMSC lysis control; In addition, AT2R was detected in exosomes derived from AT2R-overexpressing BMSC (AT2R-EXO), but not in exosomes derived from control BMSC (EXO) or exosomes derived from vehicle-control BMSC (Vehicle-EXO) (Fig. 1C). Additionally, we observed that PKH67-labelled AT2R-EXO were effectively accumulated in the cytoplasm of vascular endothelial cells (Suppl. Figure 1A).

### The Effects of AT2R-EXO on Vascular Endothelial Cells and Smooth Muscle Cells Under Hypoxia

**Fig. 2:**
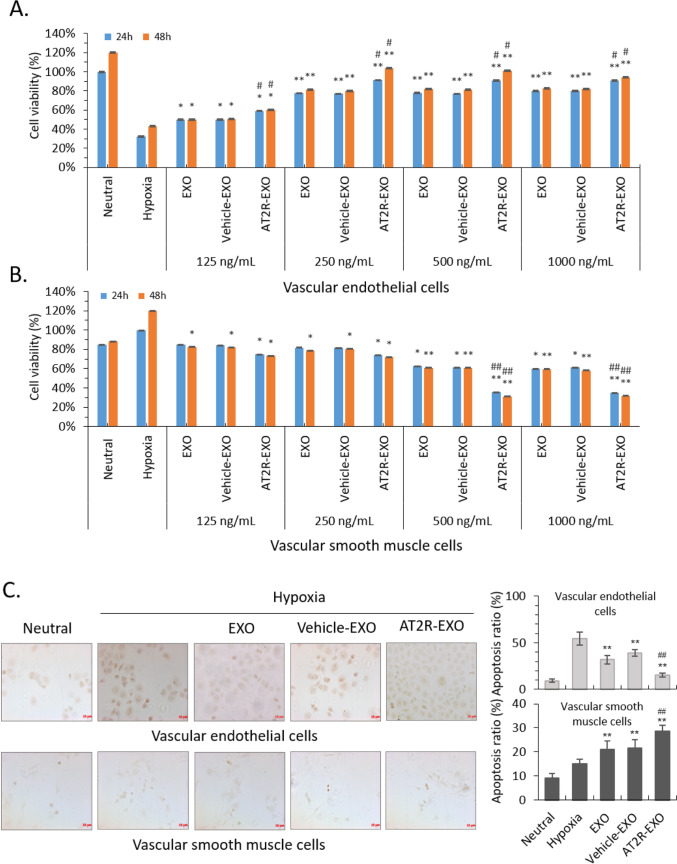

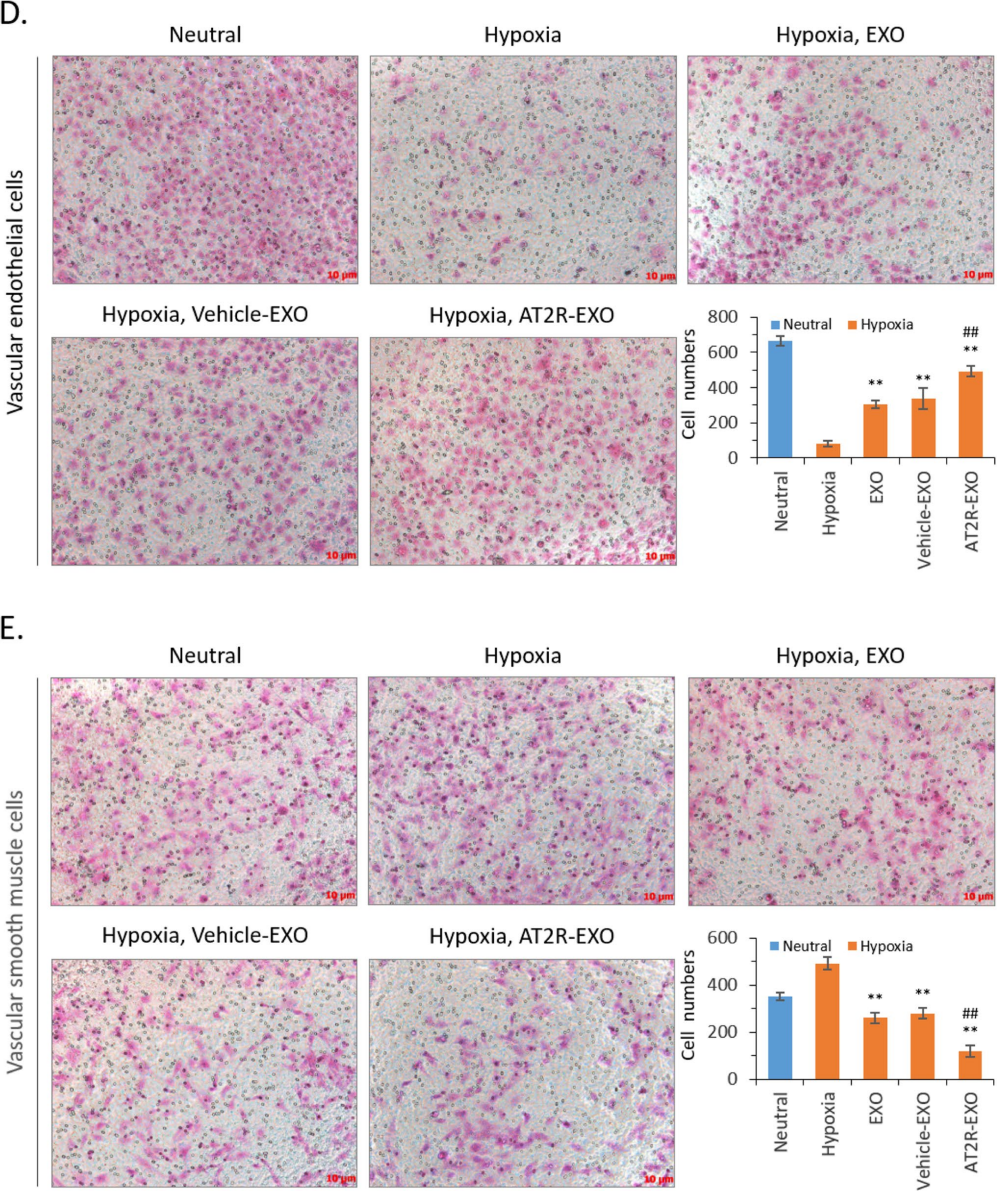
The effects of AT2R-EXO on vascular endothelial cells and smooth muscle cells under hypoxia. **A** VECs growth was analyzed by cck-8 after hypoxia induced and treated with different concentrations of the indicated exosomes for 24 h and 48 h. Data are the means ± SD from five independent experiments. *P < 0.05 and **P < 0.01 vs. hypoxia group; #*P* < 0.05 vs. vehicle-EXO group. **B** VSMCs growth was analyzed by cck-8 after hypoxia induced and treated with different concentration of the indicated exosomes for 24 h and 48 h. Data are the means ± SD from five independent experiments. *P < 0.05 and **P < 0.01 vs. hypoxia group; #*P* < 0.05 vs. vehicle-EXO group. **C** Representative images showed the result of TUNEL assay to detect the effect of exosomes on hypoxia-induced cell apoptosis. The apoptosis ratio is shown in histogram. Data are the means ± SD from three independent experiments. **P < 0.01 vs. hypoxia group; ##*P* < 0.01 vs. vehicle-EXO group. **D** Representative microphotographs showing the migration of VECs after hypoxia induced and treated with exosomes for 24 h. The migrated cell numbers are shown in histogram. Data are the means ± SD from three independent experiments. **P < 0.01 vs. hypoxia group; ##*P* < 0.01 vs. vehicle-EXO group. **E** Representative microphotographs showing the migration of VSMCs after hypoxia induced and treated with exosomes for 24 h. The migrated cell numbers are shown in histogram. Data are the means ± SD from three independent experiments. **P < 0.01 vs. hypoxia group; ##*P* < 0.01 vs. vehicle-EXO group

To further study the role of AT2R-EXO in the formation of neointima after vascular injury, we first studied the effects of AT2R-EXO on the proliferation of VECs and VSMCs under hypoxia. Our data show that all BMSC-derived exosomes had a significant proliferation-promoting effect on VECs. Compared with EXO and vehicle-EXO treatment, the AT2R-EXO treated cells had a faster proliferation rate with the best effect at a concentration of 250 ng/ml for 48 h (Fig. 2A). On the contrary, all BMSC-derived exosomes inhibited the proliferation of VSMCs, and the inhibition of AT2R-EXO at the concentration of 500 ng/mL and 1000 ng/ mL was significantly stronger than that of the EXO and vehicle-EXO control groups (Fig. 2B). Then, TUNEL assay was used to detect cell apoptosis. Our results show that exosomes can reduce hypoxia-induced apoptosis of VECs, and AT2R-EXO had a better inhibitory effect than EXO and vehicle-EXO controls. On the contrary, exosomes promoted hypoxia-induced VSMCs apoptosis, and AT2R containing exosome had the most obvious apoptotic-promoting effect (Fig. 2C). We further tested the effect of exosomes on cell migration. BMSC-derived exosomes treatment could partially alleviate the hypoxia-induced inhibitory effect of VECs; among them, AT2R-EXO had the best alleviating effect (Fig. 2D); On the contrary, hypoxia promoted the migration of VSMCs. Treatment with exosomes inhibited the migration, among them, AT2R-EXO had the best inhibitory effect (Fig. 2E).

### The Mechanism of AT2R-EXO in the Inhibition of Neointimal Hyperplasia

**Fig. 3:**
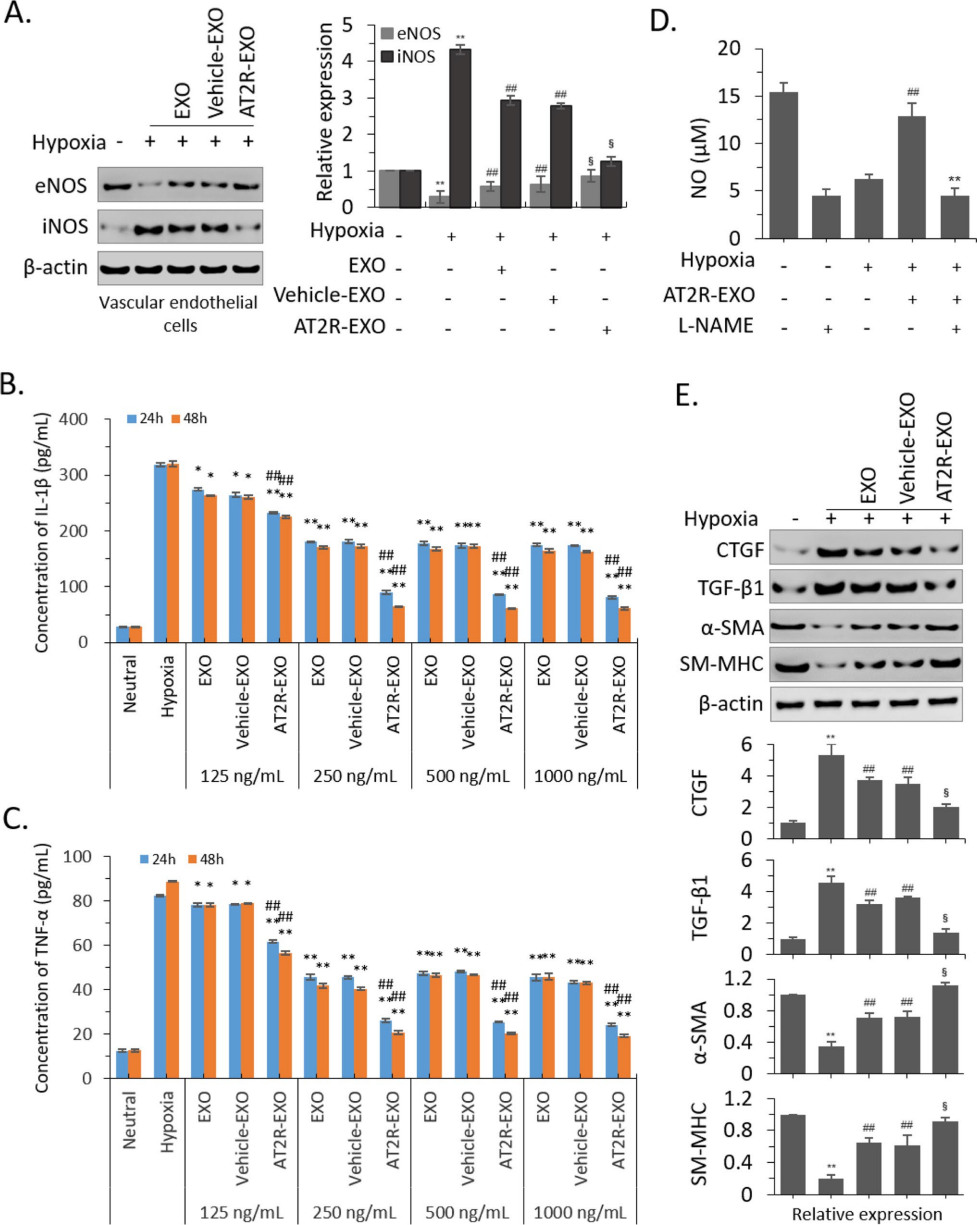
The Mechanism of AT2R-EXO in the inhibition of neointimal hyperplasia. **A** Immunoblotting analysis of the expression of eNOS and iNOS in VECs after hypoxia induced and treated with exosomes for 48 h. Relative levels were quantitatively analyzed using ImageJ (NIH). Results are the mean ± SD from three independent experiments. **P < 0.01 vs. untreated control group; ##*P* < 0.01 vs. hypoxia group; §*P* < 0.01 vs. vehicle-EXO group. **B** The secretion of IL-1β was detected by ELISA assay in VECs after hypoxia induced and treated with exosomes for 24 h and 48 h. **C** The secretion of TNF-α was detected by ELISA assay in VECs after hypoxia induced and treated with exosomes for 24 h and 48 h. Data are the means ± SD from nine independent experiments. *P < 0.05 and **P < 0.01 vs. hypoxia group; #*P* < 0.05 vs. vehicle-EXO group. **D** NO levels in VECs. Data are shown as mean ± SD of three independent experiments performed in duplicate. ##*P* < 0.01, hypoxia + AT2R-EXO treatment vs. hypoxia treatment only; **P < 0.01, hypoxia + AT2R-EXO + L-NAME treatment vs. hypoxia + AT2R-EXO treatment; **E** immunoblotting analysis of the expression of the indicated genes in VSMCs after hypoxia induced and treated with exosomes for 48 h. Relative expression levels were quantitatively analyzed using ImageJ (NIH). Results are the mean ± SD from three independent experiments. **P < 0.01 vs. untreated control group; ##*P* < 0.01 vs. hypoxia group; §*P* < 0.01 vs. vehicle-EXO group

To investigate how AT2R-EXO function in the formation of neointima, we investigated the expression of endothelial nitric oxide synthase (eNOS) and inducible nitric oxide synthase (iNOS) in VECs. Our results show that hypoxia significantly changed the ratio of eNOS/iNOS, which is manifested by a decrease in eNOS expression and an increase in iNOS expression at protein level. Treatment with BMSC-derived exosomes induced the raise of eNOS expression and partially inhibited the upregulation of iNOS, among them AT2R-EXO had the most significant effect (Fig. 3A). To further confirm the protective effect of AT2R-EXO on VECs under hypoxia, we detected the expression of downstream inflammatory factors of iNOS pathway by ELISA assay. Our results showed that hypoxia significantly induced the secretion of tumor necrosis factor α (TNF-α) and interleukin-1 β (IL-1β) in VECs. BMSC-derived exosomes treatment could inhibit hypoxia-induced the secretion, with the best inhibition effect of AT2R-EXO at a concentration of 250 ng/mL (Fig. 3B, C). Next, we detected the level of NO which is the most well-known vasodilator and regulated by eNOS. Our results showed that hypoxia significantly decreased the NO level in VECs; AT2R-EXO (250 ng/mL) could restore the level of NO, while the L-NAME (50 µM) treatment abolished the effect of AT2R-EXO (Fig. 3D). In addition, we also investigated the expression of genes related to phenotypic transformation in VSMCs. Immunoblotting showed that the expression of transforming growth factor beta 1 (TGF-β1) and its downstream connective tissue growth factor (CTGF) were upregulated under hypoxia, and the expression of α smooth muscle actin (α-SMA) and smooth muscle myosin heavy chain (SM-MHC) were significantly inhibited, while treatment with BMSC-derived exosomes significantly inhibited the alteration of those gene expression with the best inhibition effect of AT2R-EXO (Fig. 3E).

### *The Effect of AT2R-EXO on NIH After Carotid Artery Injury *In vivo

**Fig. 4:**
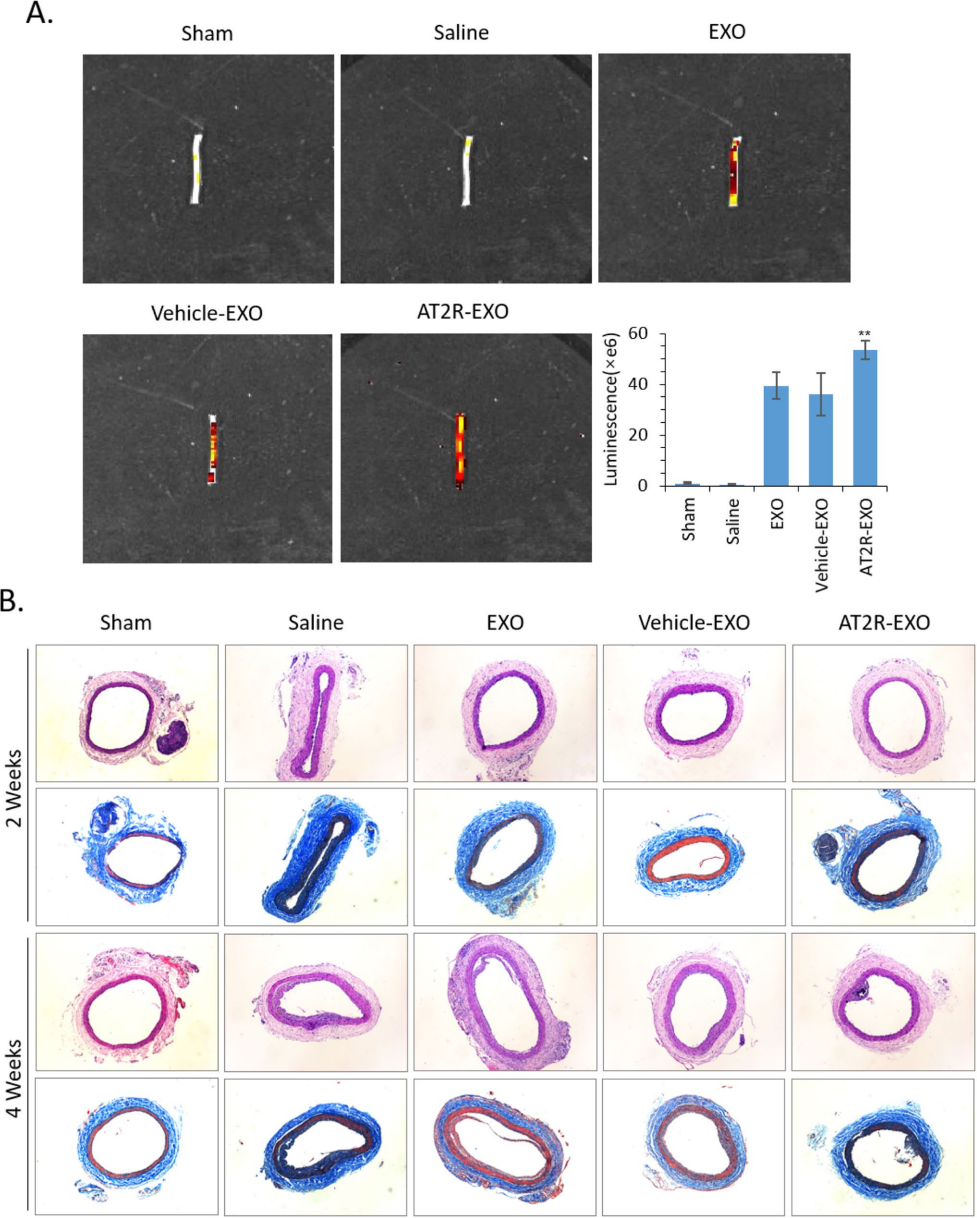

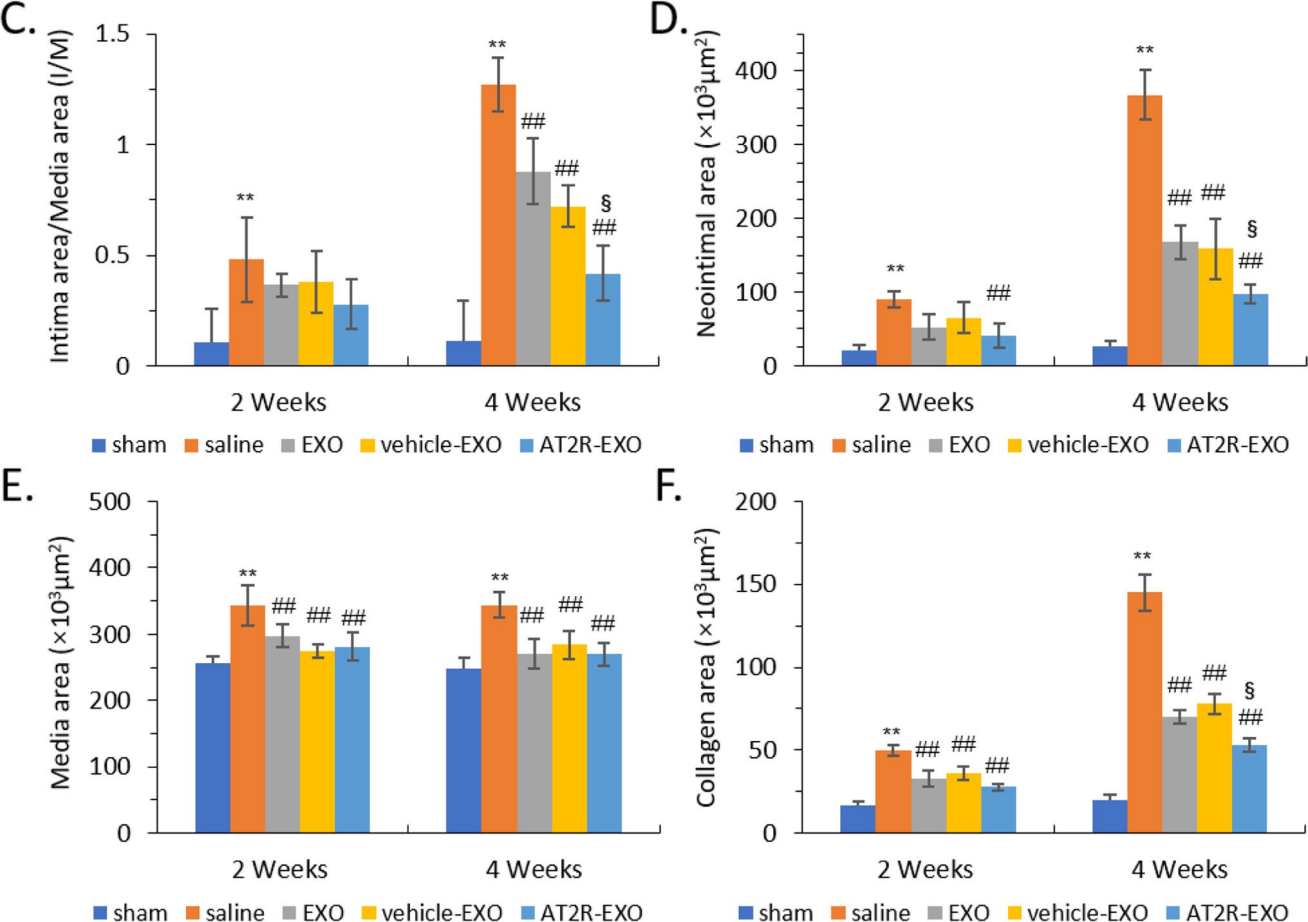
The effect of AT2R-EXO on NIH after carotid artery injury in vivo. **A** Representative intravital images showing the aggregation of exosomes in the rat carotid artery injury animal models after 2 weeks of treatment by intravenous injection. The luminescence is shown in histogram. Data are the means ± SD from five independent experiments. **P < 0.01 vs. vehicle-EXO group. **B** Representative images showing the H&E and Masson staining analysis of the formation of neointimal hyperplasia at 2 weeks and 4 weeks (*n* = 5/group, magnifications, × 40). **C** Quantitative analysis of the intimal area /media area ratio, **D** the neointimal area, **E** the media area, and **F** the collagen area of the indicated groups. Data are the means ± SD from five independent experiments. **P < 0.01 vs. sham group; ##*P* < 0.01 vs. saline group; §*P* < 0.05 vs. vehicle-EXO group

To further study the effect of AT2R-EXO on NIH after carotid artery injury in vivo, we first established a BMSC stably fusion expressing renilla luciferase-CD63 by retroviral infection (Suppl. Figure 1B). Then, after the cells were infected with AT2R-overexpression lentiviral vector or empty control lentiviral vector, we successfully extracted renilla luciferase-labeled AT2R-EXO, EXO control, and vehicle-EXO control (Suppl. Figure 1C). Next, those BMSC-derived exosomes were used to treat the rat carotid artery injury animal models by intravenous injection. After 2 weeks of treatment, we took the rat carotid artery for intravital imaging to detect the aggregation of exosomes (*n* = 5). Our results show that all BMSC-derived exosomes were found to accumulate in the injured carotid artery, and the aggregation of AT2R-EXO was better than EXO and vehicle-EXO controls (Fig. 4A). H&E and Masson staining were performed to analyze neointimal hyperplasia after 2 weeks and 4 weeks of treatment with BMSC-derived exosomes (*n* = 5) (Fig. 4B). The intimal/media (I/M) was measured as an index. Our data show that carotid artery injury increased the value of I/M compared to that in the sham group at both 2 weeks and 4 weeks. Although there was no significant reduction the I/M in the BMSC-derived exosomes groups compared to that in the saline group at 2 weeks, after 4 weeks of treatment, the I/M values of all BMSC-derived exosomes groups were significantly reduced; moreover, the reduction of AT2R-EXO group was lower than that in the EXO and vehicle-EXO control groups (Fig. 4C). The neointimal area, media area and collagen area in the BMSC-derived exosomes were all significantly reduced compared to that in the saline group at 4 weeks. Consistently, the inhibition effect of AT2R-EXO treatment on the formation of injury-induced neointimal and collagen was much more pronounced than that of EXO and vehicle-EXO treatment **(**Fig. 4D–F).

### . The Effects of AT2R-EXO on the Function of Injured Carotid Artery

**Fig. 5:**
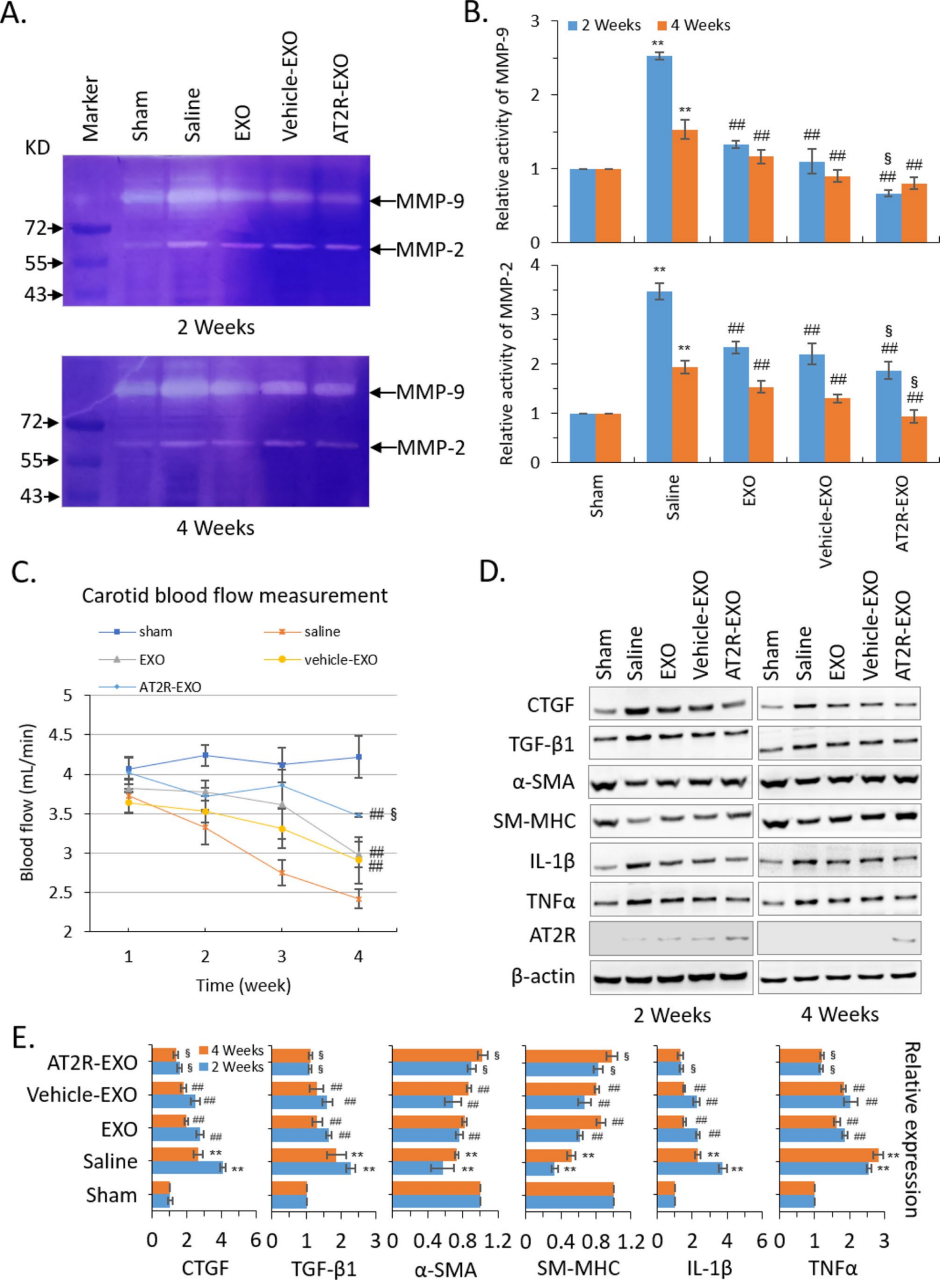
The effects of AT2R-EXO on the function of injured carotid artery. **A** Representative images showing the zymography analysis of the activity of MMP2 and MMP9 in the injured carotid artery tissue samples after treatment with exosomes for 2 weeks and 4 weeks. **B** The relative activity is shown in histogram. Data are the means ± SD from five independent experiments. **P < 0.01 vs. sham group; ##*P* < 0.01 vs. saline group; §*P* < 0.05 vs. vehicle-EXO group. **C** The spectrum Doppler detecting of the blood flow of the injured carotid artery site. Data are the means ± SD from five independent experiments. ##*P* < 0.01 vs. saline group; *P* < 0.05 vs. vehicle-EXO group. **D** Immunoblotting analysis of the expression of the indicated genes in the injured carotid artery tissue samples after treatment with exosomes for 2 weeks and 4 weeks. **E** Relative expression levels were quantitatively analyzed using ImageJ (NIH). Data are the means ± SD from three independent experiments. **P < 0.01 vs. sham group; ##*P* < 0.01 vs. saline group; §*P* < 0.05 vs. vehicle-EXO group

To further verify that systemic injection of AT2R-EXO suppressed the neointimal hyperplasia after carotid artery injury, we detected the activity of matrix metalloproteinase 2 and 9 (MMP2 and MMP9) in the injured carotid artery tissue samples (Fig. 5A). Our results show that the activities of MMP2 and MMP9 were significantly increased at 2 weeks after carotid artery injury, and treatment with BMSC-derived exosomes could partially inhibit their enhanced activities, among which the inhibition effect of AT2R-EXO was the most significant. Four weeks after injury, the activity of MMP2 and MMP9 decreased significantly compared with that at 2 weeks, and BMSC-derived exosomes treatment further promoted their activity decreasing, among which AT2R-EXO even promoted their activity back to normal level (Fig. 5B). In addition, the spectrum Doppler was used to detect the blood flow of the injured carotid artery site. Our results show that after carotid artery injury, the blood flow of each experimental group decreased gradually and reached the lowest value at the fourth week; compared with saline control group, the blood flow of the BMSC-derived exosome-treated groups was significantly higher, the treatment effect of AT2R-EXO was better with a higher blood flow than that of EXO and vehicle-EXO control groups (Fig. 5C). Further immunoblotting analysis showed that slightly expression of AT2R was detected at 2-week in all carotid artery injured groups. However, except a small amount of AT2R protein could be detected in the AT2R-EXO treatment group, AT2R was no longer detectable at 4 weeks (Fig. 5D). Moreover, BMSC-derived exosomes treatment inhibited the injury-induced upregulation of CTGF, TGF-β1, IL-1β, and TNF-α of the injured carotid artery, and enhanced the expression of α-SMA and SM-MHC; among them, AT2R-EXO had the most significant effect (Fig. 5D, E). In addition, immunofluorescence was used to examine the expression of CTGF, α-SMA, and TGF-β1. Our results show that the treatment of BMSC-derived exosomes could decrease the fluorescence intensity of CTGF and TGF-β1, while increasing the fluorescence intensity of eNOS and α-SMA compared to that in the saline group after both 2 and 4 weeks (Suppl. Figure 2).

## Discussion

Our study uses the antagonistic effects of AT1R and AT2R in the recovery of carotid arterial injury. The expression of angiotensin II (Ang II) was significantly upregulated after carotid artery injury [14, 15]. As a well-known receptor of Ang II, AT1R always maintains a high level of expression during the growth and development of the body, and its expression is further increased after vascular injury [16, 17]. The over-expressed Ang II interacts with AT1R leading to the proliferation of VSMCs, the subintimal migration, and inhibition of their apoptosis, as well as promoting the secretion of a large amount of extracellular matrix which participate in vascular remodeling and promote the occurrence and development of restenosis [18, 19]. Compared to AT1R, another receptor for Ang II and AT2R presents the negative effects [20]. The biological effects mediated by AT2R are antagonistic to the effects of AT1R on vascular tension, cell proliferation, and migration [21]. Study has shown that injection of AT2R overexpression adenovirus vector significantly reduces the neointimal hyperplasia of the carotid artery with balloon injury in rats [22]. Our previous study has also demonstrated that conditional expression of the AT2R in MSCs inhibits neointimal formation after arterial injury [8]. However, it has been reported that long-term overexpression of AT2R may lead to impairing the myocardial contractility in transgenic mice [23]. Studies have also shown that AT2R induces apoptosis in a dose-dependent manner, and moderate increasing of AT2R protects cardiac function from ischemic injury [24]. Therefore, appropriately enhancing AT2R expression in injured vessels may be beneficial to the prevention and treatment of restenosis.

AT2R is mostly expressed on the cell membrane [25, 26]. Exosomes are endocytic vesicles that are packaged by the cell membrane for substances exchange between cells [27]. Therefore, it was hypothesized that by increasing of AT2R on the cell membranes, the amount of AT2R on exosomes could be increased. In our present study, BMSCs were stably modified to overexpress AT2R with the help of lentiviral infection and the BMSC-derived exosomes were isolated. According to the results of immunoblotting, AT2R was detectable on the AT2R-EXO group, while no AT2R expression was found on the EXO and vehicle-EXO control groups. Additionally, the recipient cells, VECs and VSMCs, exhibited high uptake efficiency of those exosomes as demonstrated by a fluorescence microscopy (data not shown). These results provided foundation for the usage of exosomes in vivo and vitro.

In in vitro experiments, AT2R-EXO was able to promote the proliferation and migration of VECs and inhibit the apoptosis under hypoxia. On the contrary, AT2R-EXO was capable of inhibiting the hypoxia-induced proliferation and migration of VSMCs and promoting the hypoxia-induced apoptosis. These evidences do not only prove that BMSC-derived exosomes can promote the reendothelialization of injured artery and inhibit the phenotypic switch of VSMC [28, 29], but also indicate that AT2R can improve these effects. However, the optimal concentration of exosomes acting on VECs and VSMCs is not the same, and the difference is doubled. This may be because the exosome taken-up efficiency of these cells is different, and the taken exosomes might not fully transfer the AT2R to these cell membranes. Besides, increasing concentration of AT2R-EXO did not enhance its effectiveness, even decreased when the concentration reached 1000 ng/mL. These results indicate that the function of AT2R is closely related to its protein level [30, 31]. Compared with the continuous expression of AT2R by plasmid or lentiviral vector, the concentration and action time of AT2R can be effectively controlled through exosome delivery, which provides a new strategy for the subsequent study of AT2R and its clinical application.

Mechanistically, vascular injury is an inflammation-associated tissue damage response [32]. A large number of inflammatory mediators produced by macrophages, lymphocytes, neutrophils, and endothelial cells themselves, such as TNF-α and IL-1β can cause vascular endothelium injury [33, 34]. Our study found that BMSC-derived exosomes can significantly inhibit the secretion of TNF-α and IL-1β in hypoxia-induced VECs, while AT2R-EXO has a better inhibitory effect. However, it is not clear whether is the AT2R on the exosomal membrane or the free AT2R contained inside the exosomes that enters VECs played the roles, and the mechanism how AT2R inhibits inflammatory mediators produced by VECs needs to be further elucidated. In addition, vascular endothelial injury inevitably leads to endothelial dysfunction which is characterized by the abnormal expression of eNOS/iNOS [35, 36]. In physiological state, eNOS catalyzes arginine to produce trace amounts of NO to maintain physiological functions of blood vessels, such as vascular tension and sphincter relaxation, while the transcriptional activity of iNOS gene is relatively low. Induced by multiple inflammatory mediators, iNOS gene is activated and expressed, catalyzing arginine to synthesize a large amount of NO, which can disturb the blood pressure regulation and stimulate systemic inflammatory responses as a major inflammatory mediator [37]. Therefore, the balance of eNOS/iNOS is considered to be necessary to maintain normal function of blood vessels. Our study found that AT2R-EXO can significantly inhibit the changes of the eNOS/iNOS balance in VECs induced by hypoxia, suggesting that AT2R-EXO maintains the function of endothelial cells. However, the specific mechanism of this effect needs further study.

Phenotypic switch of VSMCs is associated in vascular diseases [38]. Synthetic phenotype of VSMCs show a sharp increase in proliferation and migration rate, and lower expression of VSMC phenotype-related markers, such as α-SMA, SM-MHC, actin-related protein smooth muscle 22α, smoothelin, calponin, and telokin [39–41]. In addition, TGF-β1 is a major cytokine that stimulates the transition of VSMCs from the contractility to synthetic phenotype [42], associating with the increase of VSMCs proliferation and migration abilities [43, 44]. Our study confirmed that hypoxia-induced VSMCs downregulated the expression of α-SMA and SM-MHC, and upregulated the expression of TGF-β1 and CTGF, while treatment with AT2R-EXO significantly inhibited the expression changes of these genes. These results suggest that AT2R-EXO can inhibit the injury-induced phenotypic transformation of VSMCs, thereby inhibiting the formation neointimal hyperplasia.

In in vivo experiments, fusion-expressed renilla luciferase-CD63 protein was used as a label for the BMSC-derived exosomes. The tetraspanin CD63 is a typical marker on the exosomal membrane [45]. It has been reported that pHluorin-CD63 was developed to study the extracellular vesicles function in zebrafish embryos vivo model [46]. pHluorin-CD63 could dynamically monitor cell migration, diffusion, and secretion of exosomes in living cells. However, due to pHluorin being a pH-sensitive GFP derivative which cannot be expressed stably, and the pHluorin’s weak fluorescence, the use of this marker is limited [47]. In our study, exosomes token cells were able to decompose luciferase substrate through the fused renilla luciferase-CD63 protein and stimulate red fluorescence. Our results show that this approach allows us to easily visualize the deposition of BMSC-derived exosomes in injured carotid artery. After treatment with BMSC-derived exosomes, H&E and Masson staining were firstly used to assess the formation of neointimal hyperplasia. Next, gelatinase assay was used to test the activity of MMP2 and MMP9 in the damaged site. Then, the spectrum Doppler was used to detect the blood flow of the injured carotid artery site. Our results of these tests show that the injection of BMSC-derived exosomes can prevent the formation of NIH in rat models, and the rats in the AT2R-EXO group had the best effect. These results suggest that AT2R might promote the positive effect of exosomes in carotid artery injury in vivo application.

## Conclusion

In carotid artery injury, on one hand, AT2R-EXO treatment promotes the proliferation and migration of VECs as well as reduces the injured-induced apoptosis resulting in accelerating of reendothelialization of injured artery. On the other hand, AT2R-EXO treatment inhibits the proliferation and migration of VMSCs as well as increases the injured-induced apoptosis, preventing the transition of VSMCs from the contractile phenotype to the synthetic phenotype. Together, AT2R-EXO prevents restenosis after carotid artery injury by attenuating the injury-induced neointimal hyperplasia.

## Supplementary Information

Below is the link to the electronic supplementary material.

Supplementary file1 (TIF 4739 KB)

Supplementary file2 (TIF 8228 KB)

## Data Availability

All data generated and/or analyzed during the present study are included in this published article.

## Abbreviations

Ang II: Angiotensin II
AT1R: Angiotensin I type receptor
AT2R: Angiotensin II type receptor
AT2R-EXO: Exosomes derived from AT2R-overexpressing BMSC
BMSCs: Bone mesenchymal stem cells
CTGF: Connective tissue growth factor
eNOS: Endothelial nitric oxide synthase
EXO: Exosomes derived from control BMSC
IL-1β: Interleukin-1 β
iNOS: Inducible nitric oxide synthase
MMP2: Matrix metalloproteinase 2
MMP9: Matrix metalloproteinase 9
NIH: Neointimal hyperplasia
TNF-α: Tumor necrosis factor α
TGF-β1: Transforming growth factor beta 1
VECs: Vascular endothelial cells
Vehicle-EXO: Exosomes derived from vehicle-control BMSC
VSMCs: Vascular smooth muscle cells
α-SMA: α Smooth muscle actin

## Declarations

### Clinical Relevance

At present, the biggest obstacle of BMSCs implantation for treatment of arterial injury is the low survival rate and high differentiation rate of BMSCs after transplantation. Also, the potential teratogenic and tumorigenic risks also significantly limit its clinical application. This study innovatively created AT2R gene-modified BMSCs exosomes (AT2R-EXO). By intravenous injection, AT2R-EXO could directionally home and aggregate to the target vascular injured area, and inhibit the formation of the neointimal hyperplasia. AT2R-EXO not only has better efficacy than EXO, but also reduces the risk of BMSCs transplantation. The findings provide new strategies for treatment of restenosis diseases after intervention.

## References

[1] M. Regmi, M.A. Siccardi, Coronary artery disease prevention, StatPearls, Treasure Island (FL), 2021. 31613540

[2] Cheng WL, She ZG, Qin JJ, Guo JH, Gong FH, Zhang P, Fang C, Tian S, Zhu XY, Gong J, Wang ZH, Huang Z, Li H (2017). Interferon regulatory factor 4 inhibits neointima formation by engaging Kruppel-like factor 4 signaling. Circulation.

[3] Stilo F, Montelione N, Calandrelli R, Distefano M, Spinelli F, Di Lazzaro V, Pilato F (2020). The management of carotid restenosis: a comprehensive review. Ann Transl Med.

[4] Waltenberger B, Liu R, Atanasov AG, Schwaiger S, Heiss EH, Dirsch VM, Stuppner H (2015). Nonprenylated xanthones from Gentiana lutea, Frasera caroliniensis, and Centaurium erythraea as novel inhibitors of vascular smooth muscle cell proliferation. Molecules.

[5] Maguire EM, Xiao Q (2020). Noncoding RNAs in vascular smooth muscle cell function and neointimal hyperplasia. FEBS J.

[6] Liles C, Li H, Veitla V, Liles JT, Murphy TA, Cunningham MW, Yu X, Kem DC (2015). AT2R autoantibodies block angiotensin II and AT1R autoantibody-induced vasoconstriction. Hypertension.

[7] Chow BS, Allen TJ (2016). Angiotensin II type 2 receptor (AT2R) in renal and cardiovascular disease. Clin Sci (Lond).

[8] Feng J, Liu JP, Miao L, He GX, Li D, Wang HD, Jing T (2014). Conditional expression of the type 2 angiotensin II receptor in mesenchymal stem cells inhibits neointimal formation after arterial injury. J Cardiovasc Transl Res.

[9] Kishore R, Khan M (2016). More Than Tiny Sacks: Stem Cell Exosomes as Cell-Free Modality for Cardiac Repair. Circ Res.

[10] Baglio SR, Rooijers K, Koppers-Lalic D, Verweij FJ, Perez Lanzon M, Zini N, Naaijkens B, Perut F, Niessen HW, Baldini N, Pegtel DM (2015). Human bone marrow- and adipose-mesenchymal stem cells secrete exosomes enriched in distinctive miRNA and tRNA species. Stem Cell Res Ther.

[11] L. Rios-Colon, E. Arthur, S. Niture, Q. Qi, J.T. Moore, D. Kumar, The role of exosomes in the crosstalk between adipocytes and liver cancer cells, Cells 9(9) (2020). 10.3390/cells9091988 PMC7563540 32872417

[12] Yan W, Li T, Yin T, Hou Z, Qu K, Wang N, Durkan C, Dong L, Qiu J, Gregersen H, Wang G (2020). M2 macrophage-derived exosomes promote the c-KIT phenotype of vascular smooth muscle cells during vascular tissue repair after intravascular stent implantation. Theranostics.

[13] Liu Z, Wu C, Zou X, Shen W, Yang J, Zhang X, Hu X, Wang H, Liao Y, Jing T (2020). Exosomes derived from mesenchymal stem cells inhibit neointimal hyperplasia by activating the Erk1/2 signalling pathway in rats. Stem Cell Res Ther.

[14] Li F, Zhang C, Schaefer S, Estes A, Malik KU (2005). ANG II-induced neointimal growth is mediated via cPLA2- and PLD2-activated Akt in balloon-injured rat carotid artery. Am J Physiol Heart Circ Physiol.

[15] Sun B, Zhao H, Li X, Yao H, Liu X, Lu Q, Wan J, Xu J (2017). Angiotensin II-accelerated vulnerability of carotid plaque in a cholesterol-fed rabbit model-assessed with magnetic resonance imaging comparing to histopathology. Saudi J Biol Sci.

[16] A.C. Montezano, A. Nguyen Dinh Cat, F.J. Rios, R.M. Touyz, Angiotensin II and vascular injury, Curr Hypertens Rep 16(6) (2014) 431. 10.1007/s11906-014-0431-2 24760441

[17] Zhang T, Yin YC, Ji X, Zhang B, Wu S, Wu XZ, Li H, Li YD, Ma YL, Wang Y, Li HT, Zhang B, Wu D (2020). AT1R knockdown confers cardioprotection against sepsis-induced myocardial injury by inhibiting the MAPK signaling pathway in rats. J Cell Biochem.

[18] Lee DY, Won KJ, Lee KP, Jung SH, Baek S, Chung HW, Choi WS, Lee HM, Lee BH, Jeon BH, Kim B (2018). Angiotensin II facilitates neointimal formation by increasing vascular smooth muscle cell migration: involvement of APE/Ref-1-mediated overexpression of sphingosine-1-phosphate receptor 1. Toxicol Appl Pharmacol.

[19] Forrester SJ, Booz GW, Sigmund CD, Coffman TM, Kawai T, Rizzo V, Scalia R, Eguchi S (2018). Angiotensin II Signal transduction: an update on mechanisms of physiology and pathophysiology. Physiol Rev.

[20] B.C. Berk, Angiotensin type 2 receptor (AT2R): a challenging twin, Sci STKE 2003(181) (2003) PE16. 10.1126/stke.2003.181.pe16 12734384

[21] F. Acconcia, The network of angiotensin receptors in breast cancer, Cells 9(6) (2020). 10.3390/cells9061336 PMC7349848 32471115

[22] Tang B, Ma S, Yang Y, Yang D, Chen J, Su X, Tan Y, Sun M, Li D (2011). Overexpression of angiotensin II type 2 receptor suppresses neointimal hyperplasia in a rat carotid arterial balloon injury model. Mol Med Rep.

[23] Bove CM, Gilson WD, Scott CD, Epstein FH, Yang Z, Dimaria JM, Berr SS, French BA, Bishop SP, Kramer CM (2005). The angiotensin II type 2 receptor and improved adjacent region function post-MI. J Cardiovasc Magn Reson.

[24] Qi Y, Li H, Shenoy V, Li Q, Wong F, Zhang L, Raizada MK, Sumners C, Katovich MJ (2012). Moderate cardiac-selective overexpression of angiotensin II type 2 receptor protects cardiac functions from ischaemic injury. Exp Physiol.

[25] Escobales N, Nunez RE, Javadov S (2019). Mitochondrial angiotensin receptors and cardioprotective pathways. Am J Physiol Heart Circ Physiol.

[26] Jang JH, Chun JN, Godo S, Wu G, Shimokawa H, Jin CZ, Jeon JH, Kim SJ, Jin ZH, Zhang YH (2015). ROS and endothelial nitric oxide synthase (eNOS)-dependent trafficking of angiotensin II type 2 receptor begets neuronal NOS in cardiac myocytes. Basic Res Cardiol.

[27] R. Kalluri, V.S. LeBleu, The biology, function, and biomedical applications of exosomes, Science 367(6478) (2020). 10.1126/science.aau6977 PMC7717626 32029601

[28] K. Liu, H. Shi, Z. Peng, X. Wu, W. Li, X. Lu, Exosomes from adipose mesenchymal stem cells overexpressing stanniocalcin-1 promote reendothelialization after carotid endarterium mechanical injury, Stem Cell Rev Rep (2021). 10.1007/s12015-021-10180-4 33982245

[29] Qu Q, Pang Y, Zhang C, Liu L, Bi Y (2020). Exosomes derived from human umbilical cord mesenchymal stem cells inhibit vein graft intimal hyperplasia and accelerate reendothelialization by enhancing endothelial function. Stem Cell Res Ther.

[30] Ohkubo N, Matsubara H, Nozawa Y, Mori Y, Murasawa S, Kijima K, Maruyama K, Masaki H, Tsutumi Y, Shibazaki Y, Iwasaka T, Inada M (1997). Angiotensin type 2 receptors are reexpressed by cardiac fibroblasts from failing myopathic hamster hearts and inhibit cell growth and fibrillar collagen metabolism. Circulation.

[31] Aranguiz-Urroz P, Soto D, Contreras A, Troncoso R, Chiong M, Montenegro J, Venegas D, Smolic C, Ayala P, Thomas WG, Lavandero S, Diaz-Araya G (2009). Differential participation of angiotensin II type 1 and 2 receptors in the regulation of cardiac cell death triggered by angiotensin II. Am J Hypertens.

[32] Inoue T, Croce K, Morooka T, Sakuma M, Node K, Simon DI (2011). Vascular inflammation and repair: implications for re-endothelialization, restenosis, and stent thrombosis. JACC Cardiovasc Interv.

[33] Hou X, Yang S, Yin J (2019). Blocking the REDD1/TXNIP axis ameliorates LPS-induced vascular endothelial cell injury through repressing oxidative stress and apoptosis. Am J Physiol Cell Physiol.

[34] Tong H, Chen R, Yin H, Shi X, Lu J, Zhang M, Yu B, Wu M, Wen Q, Su L (2016). Mesenteric Lymph duct ligation alleviating lung injury in heatstroke. Shock.

[35] Fisher M (2008). Injuries to the vascular endothelium: vascular wall and endothelial dysfunction. Rev Neurol Dis.

[36] Sodha NR, Clements RT, Sellke FW (2009). Vascular changes after cardiac surgery: role of NOS, COX, kinases, and growth factors. Front Biosci (Landmark Ed).

[37] K. Leung, [(18)F]6-(2-Fluoropropyl)-4-methyl-pyridin-2-amine, Molecular Imaging and Contrast Agent Database (MICAD), Bethesda (MD), 2004. 20641179

[38] Wang J, Li J, Cheng C, Liu S (2020). Angiotensin-converting enzyme 2 augments the effects of endothelial progenitor cells-exosomes on vascular smooth muscle cell phenotype transition. Cell Tissue Res.

[39] Shi J, Yang Y, Cheng A, Xu G, He F (2020). Metabolism of vascular smooth muscle cells in vascular diseases. Am J Physiol Heart Circ Physiol.

[40] Frismantiene A, Philippova M, Erne P, Resink TJ (2018). Smooth muscle cell-driven vascular diseases and molecular mechanisms of VSMC plasticity. Cell Signal.

[41] Liu E, Shi S, Li J, Ge R, Liang T, Li Q (2020). Farrerol maintains the contractile phenotype of VSMCs via inactivating the extracellular signal-regulated protein kinase 1/2 and p38 mitogen-activated protein kinase signaling. Mol Cell Biochem.

[42] Yao QP, Zhang P, Qi YX, Chen SG, Shen BR, Han Y, Yan ZQ, Jiang ZL (2014). The role of SIRT6 in the differentiation of vascular smooth muscle cells in response to cyclic strain. Int J Biochem Cell Biol.

[43] Wang S, Li P, Jiang G, Guan J, Chen D, Zhang X (2020). Long non-coding RNA LOC285194 inhibits proliferation and migration but promoted apoptosis in vascular smooth muscle cells via targeting miR-211/PUMA and TGF-beta1/S100A4 signal. Bioengineered.

[44] Giordano A, Romano S, Mallardo M, D’Angelillo A, Cali G, Corcione N, Ferraro P, Romano MF (2008). FK506 can activate transforming growth factor-beta signalling in vascular smooth muscle cells and promote proliferation. Cardiovasc Res.

[45] Toh WS, Lai RC, Zhang B, Lim SK (2018). MSC exosome works through a protein-based mechanism of action. Biochem Soc Trans.

[46] Verweij FJ, Revenu C, Arras G, Dingli F, Loew D, Pegtel DM, Follain G, Allio G, Goetz JG, Zimmermann P, Herbomel P, Del Bene F, Raposo G, van Niel G (2019). Live tracking of inter-organ communication by endogenous exosomes in vivo. Dev Cell.

[47] Sung BH, von Lersner A, Guerrero J, Krystofiak ES, Inman D, Pelletier R, Zijlstra A, Ponik SM, Weaver AM (2020). A live cell reporter of exosome secretion and uptake reveals pathfinding behavior of migrating cells. Nat Commun.

