# Correction to: Exosomes Derived from AT2R-Overexpressing BMSC Prevent Restenosis After Carotid Artery Injury by Attenuating the Injury-Induced Neointimal Hyperplasia

**DOI:** 10.1007/s12265-022-10308-y

**Published:** 2022-08-24

**Authors:** Xinliang Zou, Yi Liao, Zhihui Liu, Xiang Xu, Weiwei Sun, Haoran Qin, Haidong Wang, Jianping Liu, Tao Jing

**Affiliations:** 1 Department of Cardiology, Southwest Hospital, Army Medical University, Chongqing, 400038 People’s Republic of China; 2 Department of Thoracic Surgery, Southwest Hospital, Army Medical University, Chongqing, 400038 People’s Republic of China

**Correction to: Journal of Cardiovascular Translational Research**

10.1007/s12265-022-10293-2

A large portion of text comprising much of the **Discussion** section is missing from the PDF version of this published article.

Below is the **Discussion** section in its entirety.

## Discussion

Our study uses the antagonistic effects of AT1R and AT2R in the recovery of carotid arterial injury. The expression of angiotensin II (Ang II) was significantly upregulated after carotid artery injury [14, 15]. As a well-known receptor of Ang II, AT1R always maintains a high level of expression during the growth and development of the body, and its expression is further increased after vascular injury [16, 17]. The over-expressed Ang II interacts with AT1R leading to the proliferation of VSMCs, the subintimal migration, and inhibition of their apoptosis, as well as promoting the secretion of a large amount of extracellular matrix which participate in vascular remodeling and promote the occurrence and development of restenosis [18, 19]. Compared to AT1R, another receptor for Ang II and AT2R presents the negative effects [20]. The biological effects mediated by AT2R are antagonistic to the effects of AT1R on vascular tension, cell proliferation, and migration [21]. Study has shown that injection of AT2R overexpression adenovirus vector significantly reduces the neointimal hyperplasia of the carotid artery with balloon injury in rats [22]. Our previous study has also demonstrated that conditional expression of the AT2R in MSCs inhibits neointimal formation after arterial injury [8]. However, it has been reported that long-term overexpression of AT2R may lead to impairing the myocardial contractility in transgenic mice [23]. Studies have also shown that AT2R induces apoptosis in a dose-dependent manner, and moderate increasing of AT2R protects cardiac function from ischemic injury [24]. Therefore, appropriately enhancing AT2R expression in injured vessels may be beneficial to the prevention and treatment of restenosis.

AT2R is mostly expressed on the cell membrane [25, 26]. Exosomes are endocytic vesicles that are packaged by the cell membrane for substances exchange between cells [27]. Therefore, it was hypothesized that by increasing of AT2R on the cell membranes, the amount of AT2R on exosomes could be increased. In our present study, BMSCs were stably modified to overexpress AT2R with the help of lentiviral infection and the BMSC-derived exosomes were isolated. According to the results of immunoblotting, AT2R was detectable on the AT2R-EXO group, while no AT2R expression was found on the EXO and vehicle-EXO control groups. Additionally, the recipient cells, VECs and VSMCs, exhibited high uptake efficiency of those exosomes as demonstrated by a fluorescence microscopy (data not shown). These results provided foundation for the usage of exosomes in vivo and vitro.

In in vitro experiments, AT2R-EXO was able to promote the proliferation and migration of VECs and inhibit the apoptosis under hypoxia. On the contrary, AT2R-EXO was capable of inhibiting the hypoxia-induced proliferation and migration of VSMCs and promoting the hypoxia-induced apoptosis. These evidences do not only prove that BMSC-derived exosomes can promote the reendothelialization of injured artery and inhibit the phenotypic switch of VSMC [28, 29], but also indicate that AT2R can improve these effects. However, the optimal concentration of exosomes acting on VECs and VSMCs is not the same, and the difference is doubled. This may be because the exosome taken-up efficiency of these cells is different, and the taken exosomes might not fully transfer the AT2R to these cell membranes. Besides, increasing concentration of AT2R-EXO did not enhance its effectiveness, even decreased when the concentration reached 1000 ng/mL. These results indicate that the function of AT2R is closely related to its protein level [30, 31]. Compared with the continuous expression of AT2R by plasmid or lentiviral vector, the concentration and action time of AT2R can be effectively controlled through exosome delivery, which provides a new strategy for the subsequent study of AT2R and its clinical application.

Mechanistically, vascular injury is an inflammation-associated tissue damage response [32]. A large number of inflammatory mediators produced by macrophages, lymphocytes, neutrophils, and endothelial cells themselves, such as TNF-α and IL-1β can cause vascular endothelium injury [33, 34]. Our study found that BMSC-derived exosomes can significantly inhibit the secretion of TNF-α and IL-1β in hypoxia-induced VECs, while AT2R-EXO has a better inhibitory effect. However, it is not clear whether is the AT2R on the exosomal membrane or the free AT2R contained inside the exosomes that enters VECs played the roles, and the mechanism how AT2R inhibits inflammatory mediators produced by VECs needs to be further elucidated. In addition, vascular endothelial injury inevitably leads to endothelial dysfunction which is characterized by the abnormal expression of eNOS/iNOS [35, 36]. In physiological state, eNOS catalyzes arginine to produce trace amounts of NO to maintain physiological functions of blood vessels, such as vascular tension and sphincter relaxation, while the transcriptional activity of iNOS gene is relatively low. Induced by multiple inflammatory mediators, iNOS gene is activated and expressed, catalyzing arginine to synthesize a large amount of NO, which can disturb the blood pressure regulation and stimulate systemic inflammatory responses as a major inflammatory mediator [37]. Therefore, the balance of eNOS/iNOS is considered to be necessary to maintain normal function of blood vessels. Our study found that AT2R-EXO can significantly inhibit the changes of the eNOS/iNOS balance in VECs induced by hypoxia, suggesting that AT2R-EXO maintains the function of endothelial cells. However, the specific mechanism of this effect needs further study.

Phenotypic switch of VSMCs is associated in vascular diseases [38]. Synthetic phenotype of VSMCs show a sharp increase in proliferation and migration rate, and lower expression of VSMC phenotype-related markers, such as α-SMA, SM-MHC, actin-related protein smooth muscle 22α, smoothelin, calponin, and telokin [39,40,41]. In addition, TGF-β1 is a major cytokine that stimulates the transition of VSMCs from the contractility to synthetic phenotype [42], associating with the increase of VSMCs proliferation and migration abilities [43, 44]. Our study confirmed that hypoxia-induced VSMCs downregulated the expression of α-SMA and SM-MHC, and upregulated the expression of TGF-β1 and CTGF, while treatment with AT2R-EXO significantly inhibited the expression changes of these genes. These results suggest that AT2R-EXO can inhibit the injury-induced phenotypic transformation of VSMCs, thereby inhibiting the formation neointimal hyperplasia.

In in vivo experiments, fusion-expressed renilla luciferase-CD63 protein was used as a label for the BMSC-derived exosomes. The tetraspanin CD63 is a typical marker on the exosomal membrane [45]. It has been reported that pHluorin-CD63 was developed to study the extracellular vesicles function in zebrafish embryos vivo model [46]. pHluorin-CD63 could dynamically monitor cell migration, diffusion, and secretion of exosomes in living cells. However, due to pHluorin being a pH-sensitive GFP derivative which cannot be expressed stably, and the pHluorin’s weak fluorescence, the use of this marker is limited [47]. In our study, exosomes token cells were able to decompose luciferase substrate through the fused renilla luciferase-CD63 protein and stimulate red fluorescence. Our results show that this approach allows us to easily visualize the deposition of BMSC-derived exosomes in injured carotid artery. After treatment with BMSC-derived exosomes, H&E and Masson staining were firstly used to assess the formation of neointimal hyperplasia. Next, gelatinase assay was used to test the activity of MMP2 and MMP9 in the damaged site. Then, the spectrum Doppler was used to detect the blood flow of the injured carotid artery site. Our results of these tests show that the injection of BMSC-derived exosomes can prevent the formation of NIH in rat models, and the rats in the AT2R-EXO group had the best effect. These results suggest that AT2R might promote the positive effect of exosomes in carotid artery injury in vivo application.

