# Supplementary figures and images for: Exosomes Derived from AT2R-Overexpressing BMSC Prevent Restenosis After Carotid Artery Injury by Attenuating the Injury-Induced Neointimal Hyperplasia
Source: J Cardiovasc Transl Res. 2022 Jul 28;16(1):112–26. doi: 10.1007/s12265-022-10293-2 (PMC9944384; doi:10.1007/s12265-022-10293-2)

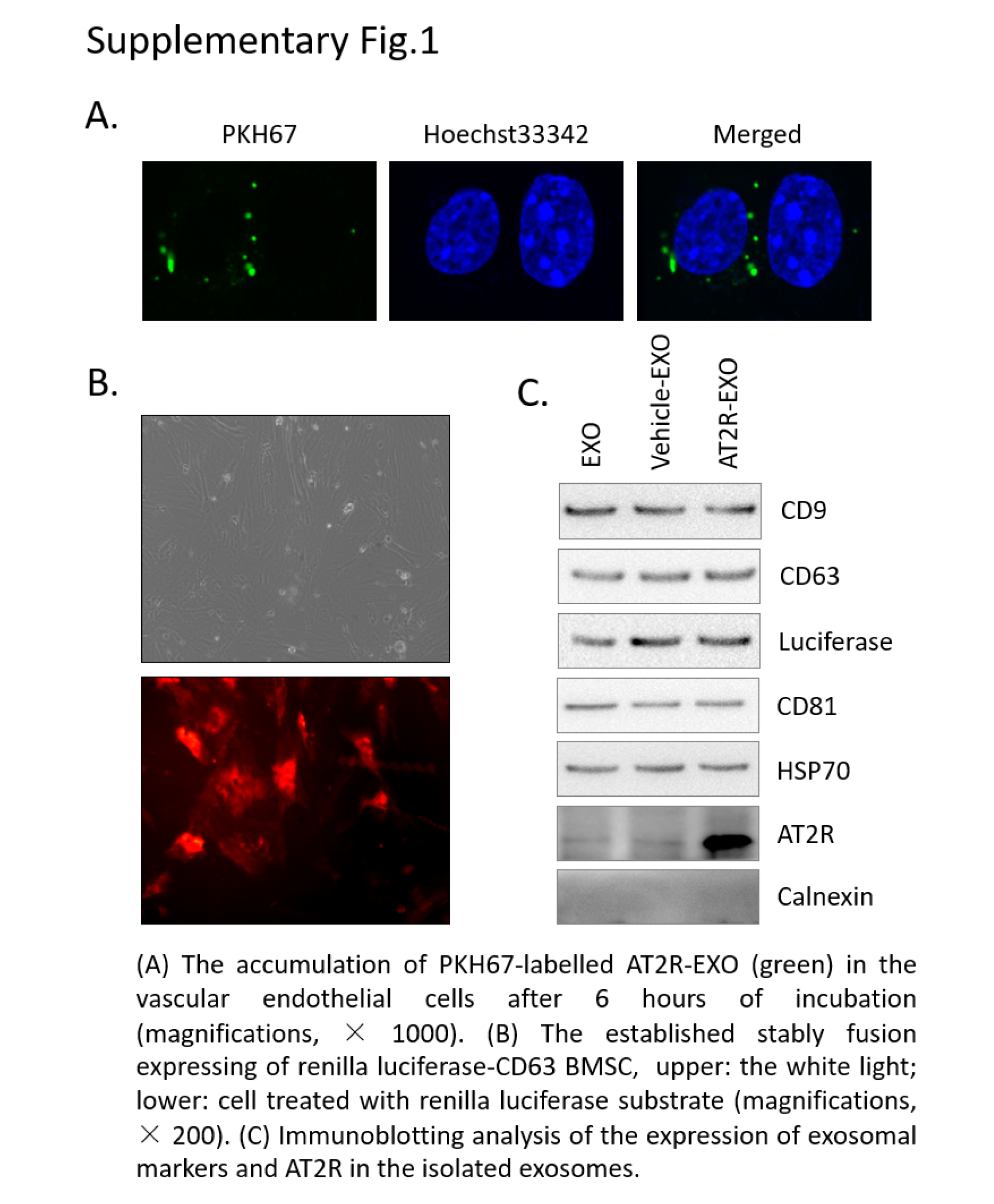

Supplement: Supplementary file 1 — Supplementary file1 (TIF 4739 KB) [file 12265_2022_10293_MOESM1_ESM.tif]

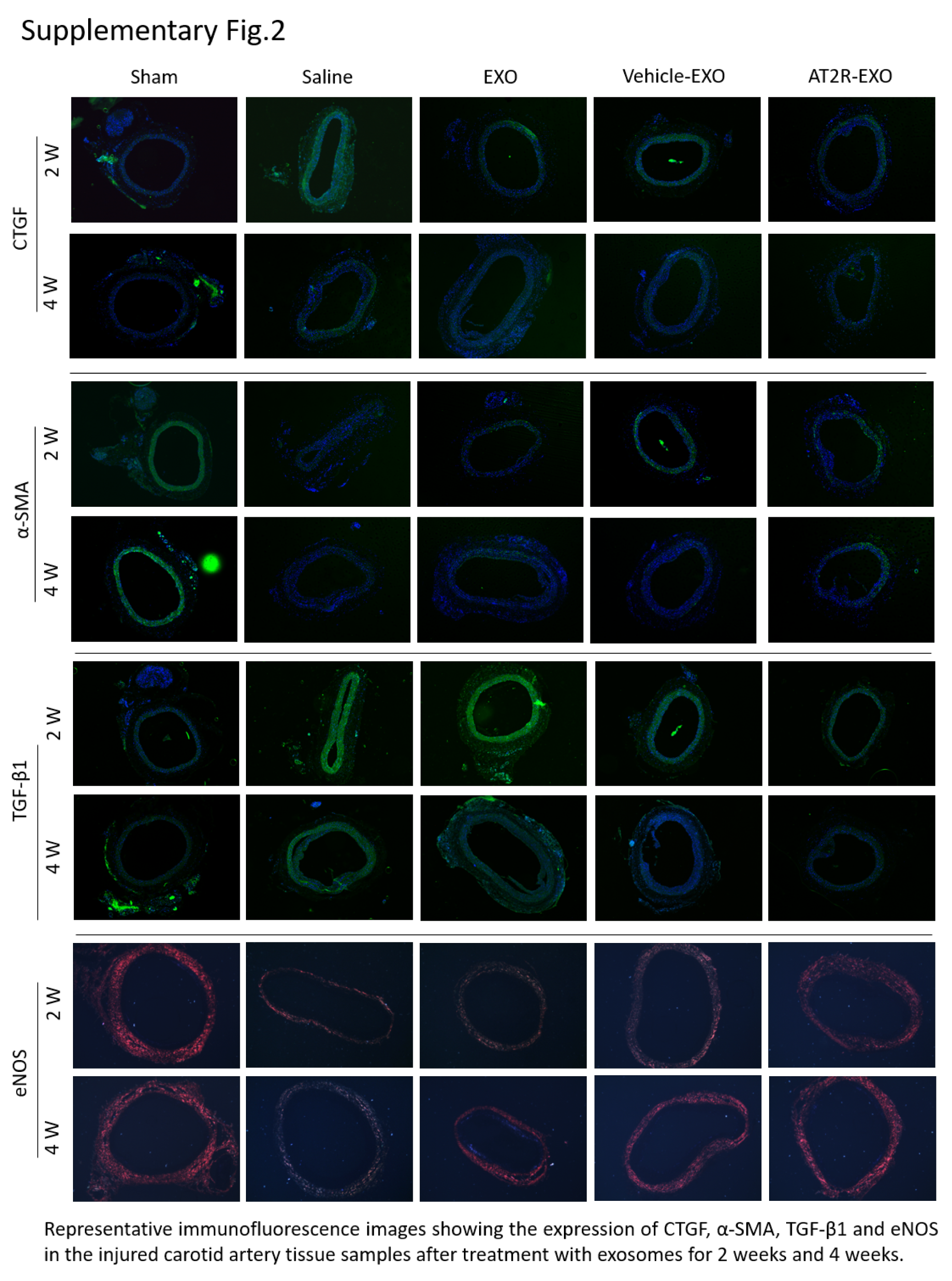

Supplement: Supplementary file 2 — Supplementary file2 (TIF 8228 KB) [file 12265_2022_10293_MOESM2_ESM.tif]
